# Understanding Workers’ Well-Being and Cognitive Load in Human-Cobot Collaboration: Systematic Review

**DOI:** 10.2196/75658

**Published:** 2025-08-27

**Authors:** Giulia Bassi, Valeria Orso, Silvia Salcuni, Luciano Gamberini

**Affiliations:** 1 Department of Developmental Psychology and Socialization University of Padua Padova Italy; 2 Human-Inspired Technology Research Center University of Padua Padova Italy; 3 Department of General Psychology University of Padua Padova Italy

**Keywords:** affective well-being, cognitive workload, operators, collaborative robots, cobots, manufacturing, Industry 5.0, systematic review

## Abstract

**Background:**

Industry 5.0 emphasizes human centricity by prioritizing human well-being alongside technological advancements. Collaborative robots (cobots) in industrial settings represent one such advancement, and their integration, particularly in manufacturing, is reshaping production processes. Although previous studies have addressed these issues, no systematic review has yet synthesized findings on how cobots impact operators’ affective well-being and cognitive workload.

**Objective:**

This study focused on psychological dimensions, which are often overlooked, particularly affective states, addressing a gap in the existing literature that has mainly emphasized the impact of cobots on the physical and cognitive workload. Specifically, we aimed to systematically review empirical studies investigating affective well-being (ie, anxiety, stress, and depression symptoms) and cognitive workload in human-cobot collaboration (HCC) within industrial settings.

**Methods:**

We conducted a comprehensive systematic search of the literature using several databases (Web of Science, Scopus, ACM Digital Library, and IEEE Xplore). Eligibility criteria included peer-reviewed empirical studies reporting quantitative or qualitative data on cognitive workload or affective well-being in HCC. Two reviewers independently conducted study selection and data extraction.

**Results:**

This review included a total of 46 studies. Findings indicated a significant increase in publications from 2020 onward, reflecting the growing interest in HCC. Most studies (28/46, 61%) were conducted in controlled laboratory settings with university students or researchers, highlighting a gap in real-world industrial research. Results indicated that, while cobots have been shown to alleviate physical fatigue and enhance job satisfaction, they also introduce new psychological challenges, including stress and anxiety symptoms due to concerns about job security and the pressures of high-paced operations. The speed at which cobots operate represents a factor affecting operators’ affective well-being and cognitive workload alongside the proximity of cobots, the system usability, and the complexity of the tasks assigned. With regard to cognitive workload, studies using physiological and self-report measures (38/46, 83%) consistently found that higher task complexity significantly raised both cognitive workload and stress levels.

**Conclusions:**

This review identified key factors that influence operators’ affective well-being and cognitive workload when working with cobots. These insights can guide the development of longitudinal research and intervention strategies, ensuring that the integration of cobots supports both productivity and operators’ well-being in manufacturing environments. To support effective implementation, future studies should be conducted in real-world settings using standardized assessment instruments, physiological measures, and qualitative interviews.

## Introduction

### Background

One of the core characteristics of Industry 5.0 is the strong emphasis on human factor and human centricity, thereby prioritizing human well-being alongside technological advancements [1]. This paradigm shift aims to enhance human-machine interactions by creating more personalized, adaptable, and inclusive work environments [2]. Industry 5.0 focuses specifically on integrating human well-being and creativity with machine efficiency, fostering a collaborative environment where humans and robots can coexist harmoniously [3]. In this direction, the integration of collaborative robots (cobots) in industrial settings, particularly in manufacturing, is reshaping the landscape of modern production processes. Cobots are designed to work alongside human operators in a shared fenceless workspace and are meant to enhance productivity, safety, and efficiency in various tasks [4]. Cobots are deployed especially in assembly tasks, where the payload capacity and accuracy typical of traditional robotic systems need to be integrated with the skills and adaptability of human operators [5]. Indeed, unlike traditional industrial robots that operate in fenced environments, cobots are built to interact directly with humans. As such, they can be viewed as a dyad, engaging in both physical and cognitive interactions [6]. This emerging human-robot collaboration (HRC) aims to blend the strengths of both parties, creating a synergistic work environment that benefits from the unique capabilities of each [7]. Studies on social robotics have evidenced that the actual experience of human-robot interaction (HRI) can influence an individual’s attitudes, perspectives, and emotional responses regarding robots [8,9]. For instance, in social and educational contexts, HRI has been shown to foster positive attitudes toward robots and support individuals’ mental well-being [10]. However, in manufacturing settings, the deployment of robots, especially cobots, appears to have mixed effects on human operators. Recent studies highlight both the potential benefits and challenges associated with cobots [11]. On the one hand, cobots can relieve human operators from repetitive tasks, reducing physical workload and improving job satisfaction [12]. They are also found to enhance job performance by enabling operators to focus on more complex and engaging activities that require human creativity and problem-solving skills [13]. However, the presence of cobots can also introduce new demands and stressors, such as the need for constant monitoring, increased cognitive load due to task complexity, and potential job insecurity [13]. The aforementioned factors can negatively impact operators’ well-being if not properly managed [14].

### State of the Art: Affective and Cognitive Well-Being Within Human-Cobot Interaction and Collaboration

As the deployment of cobots is becoming more widespread, it is essential to understand their impact on the operators’ affective well-being. According to the American Psychological Association [15], affective well-being encompasses emotional factors such as stress and depression and anxiety symptoms that may arise in operators exposed to advanced production technologies such as cobots. These factors are crucial in determining the success of the human-cobot dyad, referring to both the effectiveness of the working performance and the operators’ well-being. For example, anxiety symptoms and negative attitudes toward novel technologies can influence an individual’s trust in robots as coworkers [16]. This focus on affective well-being is particularly crucial in manufacturing, where the cognitive workload is known to be elevated [17]. Cognitive workload refers to the mental effort required during tasks, which, if excessive, can reduce job satisfaction, cause mental fatigue [18], and negatively impact operators’ affective well-being. Even though the definitions of HRI, and HRC are still blurred [19], it is essential to distinguish between the related terms often used in this field. HRI refers broadly to any form of interaction between humans and robots, encompassing both industrial and social contexts [8,9]. HRC is a subset of HRI that specifically involves cooperative task execution and goal sharing between humans and robots, often requiring a high degree of coordination [7]. Human-cobot interaction narrows the scope further to interactions with cobots, including both passive and active exchanges, without necessarily implying joint task execution. Human-cobot collaboration (HCC), which is the focus of this study, refers specifically to situations in which humans and cobots work together in a shared workspace on common tasks, combining physical or cognitive efforts [20,21]. In line with the study by Dhanda et al [20], in this review, we conceived the collaboration between humans and robots as reflected by their shared workspace and tasks. In addition, within this frame, the human-cobot dyad gains meaning not only as a task-driven entity but also as a social entity [21] that expresses itself both at an organizational and a relational level. The deployment of cobots is typically viewed primarily through the lens of productivity and safety enhancements [22]. The affective responses of workers interacting with advanced robotic systems such as cobots remain poorly understood and underreported. In this regard, previous reviews have mostly focused on the broader aspects of interaction and collaboration with industrial robots and not specifically on cobots, emphasizing cognitive and physical ergonomics over affective states. In particular, they have examined user experience [23], physical and cognitive ergonomics [24], mental workload [25], human factors [26], decision-making [27], and mental stress and safety awareness [28]. While several existing reviews have investigated HRI, ergonomics, and mental workload in industrial contexts, these works often either adopt a broad technological focus or limit their analysis to single dimensions such as physical ergonomics or user experience without specifically addressing the joint impact of cobots on both affective well-being and cognitive workload. Moreover, these reviews do not distinguish between traditional industrial robots and cobots, whose closer interaction with humans poses distinct psychological challenges. Unlike these prior works, this review uniquely synthesizes findings related to both cognitive and affective dimensions specifically within HCC, providing an integrative framework that highlights how cobots impact operators’ psychological experiences in manufacturing settings. Indeed, an overall analysis of the research addressing the effects of HCC on workers’ well-being is still missing.

Therefore, it seems relevant to conduct a systematic review of existing literature to better understand how working with cobots impacts operators’ affective well-being and how cognitive workload may contribute to these outcomes in manufacturing environments. By synthesizing the findings of the retrieved studies, this review intended to fill the identified gap in the literature, offering insights that could guide further research and the development of intervention strategies and best practices. These insights are relevant for ensuring that the technological benefits of cobots do not overshadow the organizational gains and the psychological factors of human operators, thereby aligning with the principles of Industry 5.0.

## Methods

### Search Strategy

We conducted a comprehensive search of literature published up to April 2024 using 4 electronic databases—Web of Science, Scopus, ACM Digital Library, and IEEE Xplore—to identify relevant studies. Moreover, in the initial searches, we also adopted a manual *snowballing* method to detect further relevant publications by examining references from all systematic, literature, and scoping reviews. The search aimed to include all relevant peer-reviewed articles examining the cognitive workload and affective well-being of operators working with cobots in manufacturing environments. Search terms were identified and combined using Boolean operators to ensure thoroughness. The search terms included combinations of “cobot*,” “collaborative robot*,” “human-robot interaction,” “human-cobot collaboration,” “manufacturing,” “well-being,” “stress,” “anxiety,” “depression,” “cognitive workload,” and “mental workload” (refer to Multimedia Appendix 1 for the final search strategy string).

### Selection Criteria: Inclusion and Exclusion

The inclusion criteria for selecting studies were defined as follows: (1) peer-reviewed articles published in English and for which the full text was available to the authors at the time of review (regardless of open access status); (2) studies focusing on the impact of cobots on operators’ cognitive workload in manufacturing environments; and (3) studies that provided empirical evidence on the effects of HCC on affective well-being, specifically anxiety, stress, and depression symptoms. The exclusion criteria were related to (1) studies focusing solely on technical aspects of cobots without considering the aforementioned dimensions; (2) studies that focused only on physical ergonomics related to the interaction with cobots; (3) studies not related to the manufacturing context; and (4) reviews, theoretical papers, and studies not directly examining the outlined keywords.

### Study Selection

Eligibility screening was conducted in 2 stages: initially by assessing titles and abstracts, followed by full-text review of potentially relevant studies. Two independent reviewers (GB and Giuseppe Donà) carried out the selection process. Any discrepancies were discussed and resolved by including a third reviewer (SS).

### Data Extraction and Synthesis

Data extraction was conducted independently by 2 reviewers (GB and GD). Any discrepancies in the extracted data were discussed and resolved through consensus, with the involvement of a third reviewer (SS) when necessary.

The extracted data included (1) study characteristics (title; author name; year of publication; publication type [journal or conference proceeding]; keywords; country; study objectives; study design; and sample size, mean age and SD, and gender identity); (2) the type of advanced robotic systems; (3) the type of tasks performed by the human operator and cobot; (4) the type of simulating scenarios used; (5) measures for evaluating affective well-being, in particular anxiety, stress, and depression symptoms, as well as cognitive workload; and (6) key findings and limitations of the included studies related to the purpose of this systematic review.

Due to the expected heterogeneity in study designs and outcomes, a narrative synthesis was conducted, allowing for the integration of qualitative and quantitative data. We identified key factors that impact operators’ affective well-being and cognitive workload, placing them in the broader context of HCC in manufacturing.

### Ethical Considerations

This study received approval from the ethics committee of the Human Inspired Technologies Research Center of the University of Padua, Italy (2023_212R1).

## Results

### Article Selection

**Figure 1:**
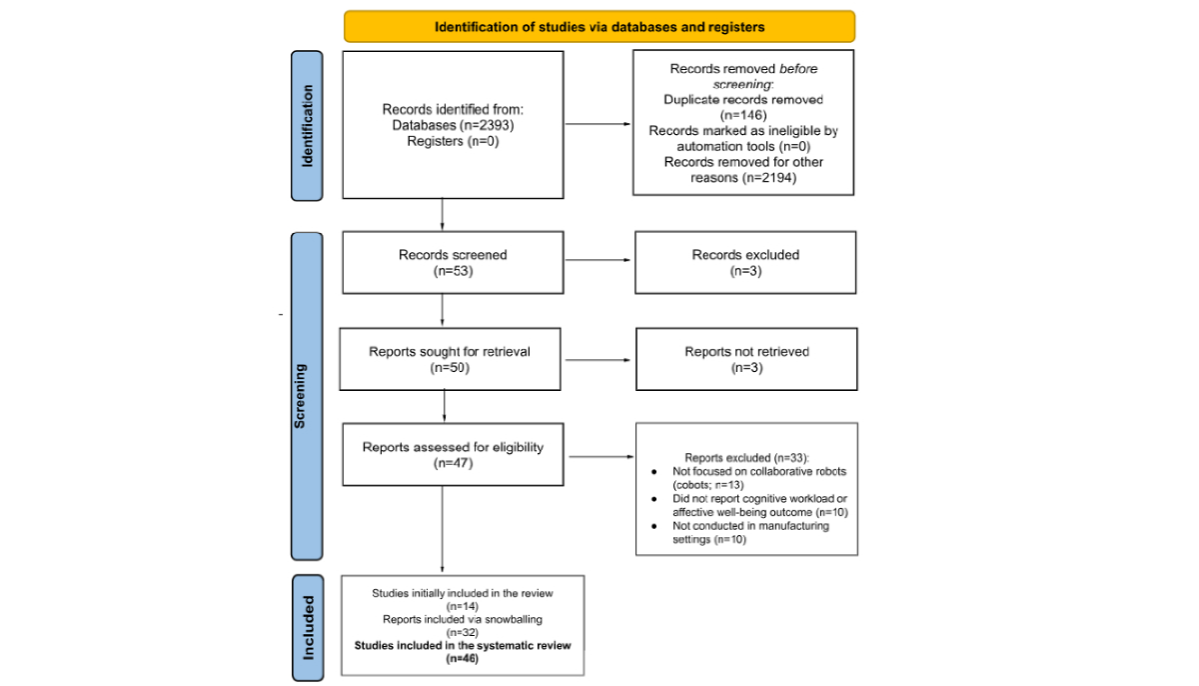
PRISMA (Preferred Reporting Items for Systematic Reviews and Meta-Analyses) flowchart.

This systematic review adhered to the PRISMA (Preferred Reporting Items for Systematic Reviews and Meta-Analyses) guidelines [29] to ensure a rigorous and transparent review process (Figure 1 [29]). The initial search yielded 2393 records. After removing duplicates (n=146, 6.1%) and records excluded for other reasons (n=2194, 91.7%), 53 records (2.21%) remained for screening. Following title and abstract screening, 3 records (5.7%) were excluded, leaving 50 reports for retrieval. Of these, 3 reports could not be retrieved, resulting in 47 reports assessed for eligibility. On the basis of the inclusion and exclusion criteria, 33 reports (70.2%) were excluded, leaving 14 studies for inclusion. Using the snowballing method, an additional 32 articles were deemed eligible, resulting in a final total of 46 articles included in the systematic review.

### Characteristics of the Included Studies

#### Advancement in the Field

Table 1 shows the number of papers published each year from 2009 to 2024. Publications increased notably since 2020. The number of articles peaked in 2023, with 24% (11/46) of the studies on HCC examining operators’ affective well-being and cognitive workload, highlighting researchers’ growing interest in this topic.

**Table 1:** The number of papers published each year from 2009 to 2024 (N=46).

Year	Studies, n (%)
2009	1 (2)
2010	2 (4)
2011	1 (2)
2016	1 (2)
2017	2 (4)
2018	3 (7)
2019	2 (4)
2020	7 (15)
2021	9 (20)
2022	5 (11)
2023	11 (24)
2024	2 (4)

**Figure 2:**
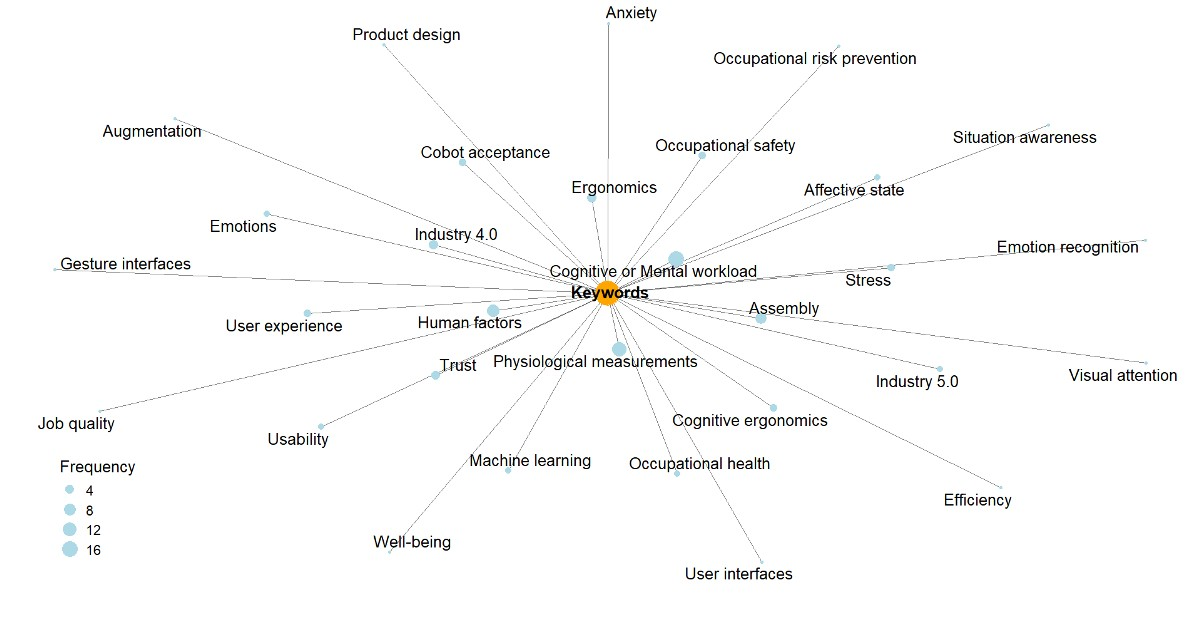
Keyword frequency network—keywords used in the included studies.

Figure 2 shows the keywords used in the included articles, creating a keyword frequency network. This network visually represents the keywords most frequently used, shedding light on the prevalent themes and areas of focus within the literature on HCC. The most frequent keywords were “cognitive or mental workload,” “physiological measurements,” “human factors,” and “occupational safety.” The frequent occurrence of these keywords suggests a significant emphasis on these dimensions, representing core areas of focus within the literature on HCC. For instance, “cognitive or mental workload” appeared often, potentially suggesting that researchers are particularly interested in understanding how human cognitive capacity and load interact with cobots. In contrast, low-frequency keywords such as “emotions,” “affective state,” “well-being,” “anxiety,” and “stress” seem to have received comparatively less attention in the literature in the context of HCC. Despite their lower frequency, these constructs are essential to understanding the human experience in HCC and can strongly influence collaboration outcomes. For example, understanding how emotions and affective states influence interaction with cobots could lead to the development of more empathetic and user-friendly technologies. Similarly, addressing issues related to anxiety and stress symptoms is essential for creating work environments that support operators’ well-being and the productivity of the organization.

#### Publication Analysis

The distribution of publications was evenly split between conference proceedings (23/46, 50%) and journal articles (23/46, 50%).

Table 2 provides a detailed breakdown of the journals in which the research papers were published, along with the number of articles published in each journal and their respective Journal Citation Reports rankings and impact factors. It shows that a large proportion of the indexed articles (9/23, 39%) fell in the second quartile, followed by 22% (5/23) of the articles in the third quartile, 17% (4/23) of the articles in the first quartile, and 9% (2/23) of the articles in the fourth quartile. Finally, 13% (3/23) of the articles were not indexed, reflecting a small portion of the total publications.

**Table 2:** Publication metrics of the included studies (N=23).

Name of the journal	Studies, n (%)	Article rank (JCR^a^)	Journal impact factor
*Computers in Human Behavior*	1 (4)	Quartile 1	9.0
*Virtual Reality*	1 (4)	Quartile 1	4.4
*Cyberpsychology, Behavior, and Social Networking*	1 (4)	Quartile 1	4.2
*International Journal of Computer Integrated Manufacturing*	1 (4)	Quartile 2	3.7
*Sensors*	2 (9)	Quartile 2	3.4
*CIRP Annals - Manufacturing Technology*	1 (4)	Quartile 2	3.2
*Applied Ergonomics*	1 (4)	Quartile 2	3.1
*Journal of Intelligent & Robotic Systems*	1 (4)	Quartile 2	3.1
*International Journal of Advanced Manufacturing Technology*	1 (4)	Quartile 2	2.9
*Frontiers in Robotics and AI*	2 (9)	Quartile 2	2.9
*Electronics*	2 (9)	Quartile 3	2.6
*Applied Sciences*	2 (9)	Quartile 3	2.5
*IISEE Transactions on Occupational Ergonomics & Human Factors*	1 (4)	Quartile 3	1.7
*Human Factors and Ergonomics in Manufacturing & Service Industries*	1 (4)	Quartile 4	2.2
*Production Engineering-Research and Development*	1 (4)	Quartile 4	1.7
arXiv	1 (4)	—^b^	—
*Procedia Manufacturing*	1 (4)	—	—
*Journal of Advanced Mechanical Design, Systems, and Manufacturing*	1 (4)	—	—

^a^JCR: Journal Citation Reports.

^b^Not available.

#### Study Characteristics

As shown in Table 3, most of the studies were conducted in Italy (15/46, 33%) and the United States (10/46, 22%). Notably, all studies were carried out in laboratories except for 4% (2/46), which took place in a real-world setting, particularly within a food company [30] and in manufacturing enterprises [31]. Across all the included studies, a total of 1031 users participated. Of these, 514 participants were unspecified regarding their professional role, 436 were researchers or students (university or graduate), and 61 were workers.

The distribution of the sample gender identity was reported in 70% (32/46) of the articles. In particular, the total sample was composed mostly of male individuals (n=535, 60%; n=350, 40% female). In addition, 61% (28/46) of the articles reported an overall mean age of 28.97 (SD 6.49) years, with an age range between 18 and 70 years.

**Table 3:** Countries in which the studies were conducted.

Country	Studies
Switzerland	[32]
United States	[33-42]
Japan	[43-45]
Germany	[46-48]
Finland	[49,50]
United Kingdom	[51-53]
Italy	[31,54-67]
France	[30,68-70]
Spain	[71,72]
Poland	[73]
Turkey	[74]
Belgium	[75]

#### Aims of the Studies and Their Theoretical Frameworks

Table 4 reports all the theoretical frameworks used in the included studies. The common goals across these studies included enhancing the interaction between humans and cobots to ensure better collaboration, understanding, and efficiency in shared tasks across various industrial manufacturing environments [32,33,43,49,54,68].

**Table 4:** Theoretical frameworks and approaches used in the included studies.

Study	Framework or approach	Main focus area	Aim or outcome
[30,60,64]	Human-centered design	Usability and user experience	Prioritize user needs in HCC^a^ design
[31]	Experience fluctuation model	Dynamic user experience	Track evolving operator experiences with cobots
[32]	Not specified (theoretical reference present)	Communication interfaces and feedback	Study effects of feedback on operator stress and performance
[49]	Task performance assessment model	Cognitive workload in dual tasks	Evaluate mental workload under complex task conditions
[54]	User control adaptation strategy	User control and speed adaptation	Analyze how adjustable cobot speed influences user fatigue
[33]	Not specified (methodological focus)	Usability and task context	Explore system usability and task interface optimization
[43]	Task modeling	Task performance	Enhance task design for improved interaction
[44]	ISO^b^ standard 10075-1 ergonomics	Mental workload and ergonomics	Apply ergonomic principles to cognitive workload in HCC
[73]	Transactional model of stress	Stress	Examine how HRI^c^ influences stress responses
[61]	TAM^d^	User acceptance	Evaluate perceived usefulness and ease of use
[74]	Extended cognition	Cognitive workload	View cognition as distributed across the human-tool system
[75]	OECD^e^ job quality and Karasek model	Job quality and stress	Assess psychosocial job conditions in HCC
[62]	Mutualistic human-machine framework	Adaptability and mutual benefit	Promote shared control and collaborative learning
[50]	PAD^f^ model	Emotional responses	Assess affective states during HRI
[40]	Grounded theory	Theory generation	Derive conceptual insights from empirical HCC data
[65]	Adaptive predictive HRI framework	Adaptation and prediction	Improve collaborative fluency
[66]	Cognitive load assessment framework	Mental demands	Evaluate and monitor cognitive load
[41]	Biobehavioral multimodal framework	Physiology, behavior, and context	Integrate real-time multimodal data in HCC
[48]	HCPS^g^	Digital-physical integration	Unify digital and physical interaction elements
[58,67]	Game-theoretic framework	Strategy and coordination	Model decision-making and joint action
[42]	RoboAssist framework	Shared control and task allocation	Facilitate adaptive human-robot cooperation

^a^HCC: human-cobot collaboration.

^b^ISO: International Organization for Standardization.

^c^HRI: human-robot interaction.

^d^TAM: technology acceptance model.

^e^OECD: Organisation for Economic Co-operation and Development.

^f^PAD: pleasure-arousal-dominance.

^g^HCPS: human-cyber-physical system.

Moreover, several studies (22/46, 48%) aimed to evaluate cognitive workload and anxiety and stress levels in human workers during interactions with cobots [34-36,44-46,51-53,55-61,69-75] while also proposing adaptive systems that can adjust cobot behaviors based on human cognitive load, stress levels, and performance [31,47,50,62].

A second group of studies (24/46, 52%) was also focused on developing frameworks and methodologies to enhance the safety, usability, and ergonomic design of HCC systems. In this regard, 48% (22/46) of the studies did not specify the frameworks used in designing their research [30,32,33,35-37,45-47,49,51-54,56,57,59,63,69-72], whereas the other 52% (24/46) of the studies detailed their framework or approach. The latter studies reported varied methodologies to enhance the efficacy and understanding of HCC.

In the included studies, approaches referred to specific methods applied to improve particular aspects of HCC. For improving user interaction, Memar and Esfahani [34] used a subject-independent feature selection approach to enhance generalizability across different users. Arai et al [44] applied the International Organization for Standardization 10075-1 standard approach to address ergonomic principles related to mental workload. Pollak et al [73] adopted a transactional approach to stress symptoms, examining how HRI influences stress responses. Focusing on human-centered design, other studies (3/46, 7%) [30,60,64], prioritized the needs and experiences of users, whereas the approach developed by Sadrfaridpour and Wang [38] and Sadrfaridpour et al [39] integrated a physical and social HRC framework to further promote the holistic understanding of interaction dynamics. In addition, Eyam et al [50] introduced a psychological dimension by using the pleasure-arousal-dominance approach to measure emotional responses in HRI. Further contributions to the current field include the study by Storm et al [31], who captured dynamic changes in user experiences using the experience fluctuation model. Moreover, Tan et al [43] used a task modeling approach to enhance task performance in interactions, and Ustunel and Gunduz [74] adopted an extended cognition approach, viewing cognitive processes as extending beyond the individual to include tools and environments. Finally, Zhao et al [40] applied the grounded theory approach to derive theoretical insights from empirical data.

With regard to the development of frameworks, which are comprehensive, structured systems designed to provide solutions and structures for HCC, the study by Lagomarsino et al [66] proposed a cognitive load assessment framework to evaluate the mental demands placed on users. In their subsequent study [65], they introduced an HRI framework incorporating adaptive and predictive models to enhance collaboration efficiency. In this direction, Bettoni et al [62] contributed by developing a mutualistic and adaptive human-machine collaboration framework emphasizing adaptability and mutual benefit. Recent multimodal frameworks include the studies by Hopko et al [41], who developed a biobehavioral framework integrating physiological, behavioral, and contextual data, and the study by Kalatzis et al [68], who combined visual, auditory, and tactile data to improve interaction quality and robustness. Moreover, Pantano et al [48] integrated physical and digital aspects through a human-cyber-physical system framework. In terms of degrees of collaboration, Messeri et al [67] used a game-theoretic framework to model and analyze interactions focusing on strategic decision-making processes between agents. Subsequently, Messeri et al [58] emphasized collaborative methods such as joint planning and coordinated task execution, highlighting their integration within the game-theoretic framework. Rajavenkatanarayanan et al [42] proposed the RoboAssist framework, which includes methods such as shared control and adaptive task allocation to facilitate human-robot cooperation. Rossato et al [61] applied the technology acceptance model framework to assess factors influencing user acceptance of robots, relying on collaborative methods such as user feedback loops and iterative design to enhance user interaction and acceptance. Finally, Van Dijk et al [75] used the Organisation for Economic Co-operation and Development job quality framework and the Karasek job-demand-control model to assess job quality in the context of HCC.

#### Description of the Advanced Robotic Systems Used in the Included Studies

When exploring the technology used in manufacturing processes, we found that only 2% (1/46) of the studies [74] did not specify the robotic technology used, and 4% (2/46) of the studies used cobots in real-world settings [30,31]. Table 5 illustrates the distribution of the technologies used.

**Table 5:** Distribution of the technologies used in descending order.

Technology used	Studies, n (%)
Universal Robots	9 (50)
Mobile manipulator cobots	7 (15)
ABB YuMi cobot	6 (13)
Baxter robot	5 (11)
Franka Emika Panda robot with a robotic collaborative gripper	2 (3)
REIS R 30-16 industrial cobot	1 (2)
Sawyer cobot	1 (2)
S Powerball LWA 4P robot arm	1 (2)

A large proportion of the studies (18/46, 39%) relied on cobots from Universal Robots with different weight categories. Notably, 50% (9/18) of these studies explored collaborative tasks using a UR3 cobot [30,40,54,56,57,59,63,68,71,72]. A total of 17% (3/18) of the studies used a UR5 cobot [36,62,64]. In particular, the study by Nenna et al [64] realistically reproduced a robotic arm through virtual reality (VR), and the study by Luo et al [36] incorporated a Fetch Freight Base (Fetch Robotics, Inc) as the specific robot platform. One study [53] used both UR3 and UR5 cobots. Furthermore, 11% (2/18) of the studies used the UR10 cobot [35,41], and another 11% (2/18) used the UR10e model [60,61]. Finally, Lagomarsino et al [65] used the UR16e cobot equipped with Robotiq’s vacuum gripper EPick.

In addition, 11% (5/46) of the studies used the Baxter robot [33,37-39,51].

Another 13% (6/46) of the studies used the ABB YuMi cobot [49,50,58,67,70,75]. Furthermore, 9% (4/46) of the studies used the KUKA LBR iiwa R800 cobot [47,48,52,73], whereas 2% (1/46) of the studies [32] used 2 KUKA LWR IV+ robotic arms, each equipped with joint torque sensors at the actuators and a 6-axis ATI Industrial Automation force and torque sensor on the end effector, with a 3D-printed flat palm tool for grasping tasks.

Moreover, 9% (4/46) of the studies focused on mobile manipulator cobots. Specifically, Dehais et al [69] used the Jido mobile manipulator cobot featuring an MP-L655 platform from Neobotix and a 6-df Mitsubishi PA-10 arm equipped with various sensors, including sonars, laser range finders, stereo cameras, contact sensors, and a wrist force sensor. Similarly, 7% (3/46) of the studies [43-45] involved prototypes of mobile robots with 2 manipulators similar in size to human arms designed for assisting human operators in cell production assembly tasks.

Furthermore, 9% (4/46) of the studies used different robotic systems. In particular, Rajavenkatanarayanan et al [42] used the Sawyer cobot by Rethink Robotics as part of the RoboAssist system to monitor the cognitive load of human teammates during collaborative assembly tasks. Moreover, Memar and Esfahani [34] used a 6-df lightweight SCHUNK Powerball LWA 4P robot arm equipped with a 6-axis force and torque sensor for implementing admittance control in HRI. Finally, Koppenborg et al [46] used the REIS R 30-16 industrial cobot, whereas Lagomarsino et al [65,66] used the Franka Emika Panda robot with a Robotiq collaborative gripper for a collaborative assembly task assessing cognitive ergonomics in HCC.

#### Description of the Specific Tasks Performed by the Human and Cobot

The included studies used a wide range of human-cobot tasks, focusing particularly on assisting and increasing human performance in various assembly, manipulation, and interactive settings within the context of manufacturing.

The most used tasks included assembly and disassembly, comprising various components such as pneumatic cylinders, mechanical parts, and industrial components [30,31,37-41,43-45,47,54,56-58,60-63,65-68,70-73,75].

Another category involved picking and placing, sorting, holding, and positioning parts [32,36,42,43,45,48,50,64,74], as well as performing repetitive movements to bring parts into a shared workspace [49,59,69].

Feeding parts, extracting, and handling materials were also common tasks [37,52], along with comanipulation of objects, polishing tasks, and assisting with gross and fine manipulation tasks [33-35].

Finally, tasks involving information acquisition, processing, and decision-making were also used [30,46,51,53].

#### Description of the Simulating Scenarios Developed in the Included Studies

Except for the 4% (2/46) of the studies conducted in a real-world setting [30,31] and one study that did not report a description of the simulation scenario [74], 93% (43/46) of the studies used experimental scenario tasks to investigate HCC, focusing on actual industrial and manufacturing scenarios to ensure the relevance and applicability of their findings.

#### Exploring HCC in Construction Experimental Scenario Tasks

In the study by Baxter et al [51], participants sorted blocks based on visual cues with cobot assistance, which intervened in after 5 seconds of inactivity. Chacón et al [71] simulated the Tower of Hanoi task with and without cobot collaboration. Fournier et al [70] divided participants into solo and cobot-assisted groups for an industrial assembly task using Duplos, large interlocking building bricks commonly used in prototyping and simulation tasks. In the study by Eyam et al [50], participants and a cobot assembled a wooden box, adapting cobot actions based on real-time electroencephalography (EEG) data. Messeri et al [58,67] explored stress, productivity, and role dynamics in collaborative tasks, adjusting cobot interactions accordingly. Finally, Zhao et al [40] investigated task interdependence levels in building toy houses with cobots.

#### Exploring HCC Through Specific Experimental Scenario Tasks

Gervasi et al [63] compared manual assembly versus cobot-assisted assembly of tile cutters, whereas Mariscal et al [72] contrasted assembly tasks performed with a human partner with a cobot delivering parts. Brunzini et al [55] created scenarios with cognitive workloads through time pressure, dual tasks, and standard conditions in tractor engine maintenance. Similarly, Kalatzis et al [68] varied cognitive workload and user interface (UI) conditions (no interface, display UI, and mixed reality UI) in a pick-and-place task with a cobot. Hopko et al [35] used scenarios with different levels of robotic assistance and cognitive fatigue for metal polishing tasks, and they also assessed reliable and unreliable cobot performance in assembly tasks [41]. In this regard, Memar and Esfahani [34] tested scenarios with varying damping levels (low, medium, and high) in cobot-assisted tasks. Lagomarsino et al [65,66] involved a cobot with a vacuum gripper in box-filling tasks, whereas Rajavenkatanarayanan et al [42] divided the assembly of a miniature sanding machine between humans and cobots. Luo et al [36] implemented retail scenarios in which cobots assisted with tasks such as cart pushing and sorting, whereas Eimontaite et al [52] used scenarios focusing on bolt extraction in a manufacturing-type environment. Moreover, Rahman [37] used affect-based motion planning for task allocation between humans and cobots. Finally, Sadrfaridpour and Wang [38] and Sadrfaridpour et al [39] explored hybrid manufacturing cell scenarios with cobots delivering parts for assembly, and Bettoni et al [62] simulated an injection molding line with baseline and adaptive cobot assistance.

#### Exploring HCC Through VR Experimental Scenarios

Koppenborg et al [46] examined the impact of cobot movement speed and predictability on human operators within a VR environment designed to mimic an industrial workplace. Participants completed tasks while cobots moved at either 750 or 1400 mm/s, with movement patterns being either regular or erratic. Nenna et al [64] explored the potential of VR simulations within HRC frameworks. Using a head-mounted display, they replicated physical pick-and-place tasks in a virtual environment, focusing on the effectiveness and realism of these simulations. In addition, Arntz et al [47] simulated HCC in VR through 2 scenarios. The baseline condition involved a nonaugmented robot arm, whereas the augmented condition featured a cobot that provided guidance using text panels, light signals, and gestures.

#### Exploring HCC Through Hazard Experimental Scenarios

One study [48] involved 3 types of simulated hazards: collision, squeeze, and workpiece drop. Researchers simulated collision and squeeze hazards by entering the danger zone and obstructing the cobot’s movement, whereas the workpiece drop was simulated by attempting to open the gripper during a task.

#### Exploring HCC Through Various Speeds, Gestures, and Types of Communication in Experimental Scenarios

Dehais et al [69] evaluated human-cobot handover tasks with different motion plans and velocities. Gervasi et al [56] varied cobot movement speed, operator distance, and task execution control. Fujita et al [45] studied mental strain with different cobot speeds, motion trajectories, and preknowledge of cobot speed. Fraboni et al [54] investigated assembly tasks in a Smart Mini Factory laboratory with 3 autonomy levels. Panchetti et al [59] examined assembly tasks with varying cobot speed, autonomy, trajectory planning, UIs, and safety notifications. Van Dijk et al [75] assessed workload impact based on human autonomy and cobot work pace during collaborative tasks. Gualtieri et al [57] explored cognitive ergonomics through scenarios of low, medium, and high interaction levels. Amanhound et al [32] evaluated control strategies to enhance industrial assembly tasks. Lagomarsino et al [66] compared human-human collaboration, HRI, and HRC in assembly tasks. Zakeri et al [53] simulated multitasking in a collaborative factory setting. Arai et al [44] studied operator stress levels by varying cobot distance, speed, and movement notice. Tan et al [43] focused on a collaboration prototype for cable harness assembly, identifying key design factors. Lemasurier et al [33] tested intent-signaling methods in shared workspaces via motion- and light-based signals. Finally, Aromaa et al [49] evaluated gesture-based interfaces in industrial contexts using digital human models.

#### Methods Used

##### Overview

Table 6 shows the specific measures used in the included studies. Several studies (17/46, 37%) incorporated both physiological and self-report measures to provide a comprehensive assessment of HCC. Moreover, 35% (16/46) of the studies used only self-report measures (eg, questionnaires), and 22% (10/46) of the studies relied only on physiological measures. Finally, 4% (2/46) of the studies used semistructured interviews to collect qualitative data regarding participants’ emotional states.

**Table 6:** Detailed measures used in the included studies.

Study	Construct	Physiological or behavioral measures	Self-report tools
Brun and Wioland [30]	Stress	—^a^	Interviews
Storm et al [31]	Cognitive workload	HRV^b^	—
Amanhound et al [32]	Cognitive workload	—	NASA-TLX^c^
Aromaa et al [49]	Cognitive workload	—	NASA-TLX
Fraboni et al [54]	Cognitive workload	—	Single item from the NASA-TLX
Kalatzis et al [68]	Stress and cognitive workload	Stress: HRV; cognitive workload: —	Stress: —; cognitive workload: SART^d^
Lemasurier et al [33]	Cognitive workload	—	NASA-TLX
Tan et al [43]	Cognitive workload	SPR^e^	—
Memar and Esfahani [34]	Anxiety	EEG^f^	—
Arai et al [44]	Stress	SPR	Semantic differential questionnaire
Baxter et al [51]	Cognitive workload	—	NASA-TLX
Brunzini et al [55]	Cognitive workload and stress	—	Cognitive workload: NASA-TLX; stress: NAS^g^
Chacón et al [71]	Cognitive workload	—	NASA-TLX
Dehais et al [69]	Stress	GSR^h^	VAS^i^
Eimontaite et al [52]	Anxiety	Facial expression analysis	RAS^j^
Fournier et al [70]	Cognitive workload	—	NASA-TLX
Fujita et al [45]	Stress	SPR	Semantic differential questionnaire devised ad hoc
Gervasi et al [56]	Stress	SCRs^k^	SAM^l^
Gualtieri et al [57]	Stress and cognitive workload	—	Questionnaire devised ad hoc, interviews, and observations
Koppenborg et al [46]	Stress, anxiety, and cognitive workload	—	SAM, STAI-S^m^, and NASA-TLX
Hopko et al [35]	Stress and cognitive workload	Stress: HRV; cognitive workload: —	Stress: —; cognitive workload: SART and NASA-TLX
Luo et al [36]	Cognitive workload	Eye and pupil tracking	—
Mariscal et al [72]	Cognitive workload	Eye tracking	NASA-TLX
Messeri et al [58]	Stress and anxiety	—	PANAS^n^ and STAI^o^
Panchetti et al [59]	Stress and cognitive workload	Stress: —; cognitive workload: eye tracking	Stress: Short Stress State Questionnaire; cognitive workload: NASA-TLX
Pollak et al [73]	Stress	HRV	PASA^p^
Pluchino et al [60]	Cognitive workload	Eye tracking and HRV	NASA-TLX
Rossato et al [61]	Cognitive workload	—	NASA-TLX (frustration subscale)
Ustunel and Gunduz [74]	Cognitive workload	—	NASA-TLX
Van Dijk et al [75]	Cognitive workload	—	NASA-TLX
Zakeri et al [53]	Anxiety and cognitive workload	Anxiety: EEG; cognitive workload: EEG and fNIRS^q^	Anxiety: —; cognitive workload: NASA-TLX
Arntz et al [47]	Stress and cognitive workload	—	Stress: PSS^r^; cognitive workload: NASA-TLX (frustration subscale)
Bettoni et al [62]	Stress and cognitive workload	HRV, electrodermal activity, and skin temperature	Questionnaire adapted from the NASA-TLX
Eyam et al [50]	Stress	EEG	—
Gervasi et al [63]	Stress	HRV and SCL^s^	—
Rahman [37]	Cognitive workload	—	NASA-TLX
Nenna et al [64]	Cognitive workload	Eye tracking	NASA-TLX
Sadrfaridpour and Wang [38]	Cognitive workload	—	NASA-TLX
Sadrfaridpour et al [39]	Cognitive workload	—	NASA-TLX
Zhao et al [40]	Stress and anxiety	HRV	—
Lagomarsino et al [65]	Cognitive workload	HRV	—
Lagomarsino et al [66]	Stress and cognitive workload	GSR, SCL, and SCR	Stress: —; cognitive workload: NASA-TLX
Hopko et al [41]	Cognitive workload	fNIRS	NASA-TLX
Pantano et al [48]	Cognitive workload	—	NASA-TLX
Messeri et al [67]	Stress	HRV	—
Rajavenkatanarayanan et al [42]	Stress and anxiety	SCL and EEG	—

^a^Not applicable.

^b^HRV: heart rate variability.

^c^NASA-TLX: NASA Task Load Index.

^d^SART: Situation Awareness Rating Technique.

^e^SPR: skin potential response.

^f^EEG: electroencephalography.

^g^NAS: numerical analogue scale.

^h^GSR: galvanic skin response.

^i^VAS: visual analogue scale.

^j^RAS: Robot Anxiety Scale.

^k^SCR: skin conductance response.

^l^SAM: Self-Assessment Manikin.

^m^STAI-S: short version of the State-Trait Anxiety Inventory.

^n^PANAS: Positive and Negative Affect Schedule.

^o^STAI: State-Trait Anxiety Inventory.

^p^PASA: Primary Appraisal Secondary Appraisal.

^q^fNIRS: functional near-infrared spectroscopy.

^r^PSS: Perceived Stress Scale.

^s^SCL: skin conductance level.

##### Tools Used for Assessing Affective Well-Being

#### Overview

Table 7 reports the distribution of self-report tools and the physiological measures used for assessing affective well-being.

**Table 7:** Measures used for assessing operators’ affective well-being.

Measure		Studies, n (%)
**Self-report measures**		
	Numerical analogue scale	1 (2)
	Perceived Stress Scale	1 (2)
	Positive and Negative Affect Schedule	1 (2)
	Robot Anxiety Scale	1 (2)
	Self-Assessment Manikin	2 (4)
	Semantic differential questionnaire	2 (4)
	Short Stress State Questionnaire	1 (2)
	State-Trait Anxiety Inventory	2 (4)
	Visual analogue scale	1 (2)
**Physiological measures**		
	EDA^a^	5 (11)
	EEG^b^	3 (7)
	HRV^c^	6 (13)

^a^EDA: electrodermal activity.

^b^EEG: electroencephalography.

^c^HRV: heart rate variability.

#### Self-Report Tools Used for Assessing Affective Well-Being

Several studies (10/46, 22%) used various self-report tools to evaluate affective well-being. Most of them (5/10, 50%) investigated the perceived stress as a proxy of users’ well-being. In particular, Arntz et al [47] used the Perceived Stress Scale (PSS) after the exposure to specific narrative scenarios presented as text. The PSS is a unidimensional measure composed of 10 items rated on a 5-point Likert scale designed to measure stress levels in the previous month. Arai et al [44] and Fujita et al [45] used a semantic differential questionnaire to evaluate psychological states such as fear, surprise, and discomfort on a scale from 0.0 to 6.0, with participants rating their feelings immediately after each trial of cobot motions. Panchetti et al [59] assessed perceived stress through the Short Stress State Questionnaire, which is a short version of the Dundee Stress State Questionnaire. The Short Stress State Questionnaire consists of 24 items evaluating 3 dimensions—task engagement, distress, and worry—on a 5-point Likert scale. Pollak et al [73] used the Primary Appraisal Secondary Appraisal instrument to measure stress appraisal. More specifically, the Primary Appraisal Secondary Appraisal scale includes 16 items, which are divided into two main categories: (1) Primary Appraisal (8 items) evaluates whether an event or situation poses a threat to an individual’s well-being, and (2) Secondary Appraisal (8 items) assesses an individual’s ability to cope with the perceived stressful event. Finally, Brunzini et al [55] administered the numerical analogue scale to evaluate perceived stress. The scale has been adapted and used in various studies within the workplace [76,77]. The numerical analogue scale is characterized by a single item rated through a numerical rating system ranging from 0 to 10 or 0 to 100, where 0 indicates no intensity of perceived stress symptoms and the highest number indicates maximum intensity of perceived stress symptoms. Similarly, Dehais et al [69] used the visual analogue scale to detect perceived stress symptoms. This scale consists of 1 item, and being a continuous scale, it is usually represented as a horizontal line ranging from 0 to 100 mm. One end indicates no intensity of perceived stress symptoms, whereas the other end indicates maximum intensity of perceived stress symptoms. Moreover, 4% (2/46) of the studies [46,58] evaluated anxiety symptoms using the State-Trait Anxiety Inventory in its long and brief versions. The State-Trait Anxiety Inventory consists of 20 items (long version) and 6 items (short version) rated on a 4-point Likert scale evaluating both trait anxiety (the general and long-standing propensity to experience anxiety) and state anxiety (the level of anxiety experienced in a given moment). Another study [52] evaluated anxiety symptoms related to the interaction with cobots by relying on the Robot Anxiety Scale. This unidimensional scale consists of 18 items rated on a 5-point Likert scale. It assesses various dimensions of anxiety, such as fear of robots, discomfort in interacting with robots, and the perceived threat posed by robots.

A total of 7% (3/46) of the studies used measures to evaluate overall affective well-being. In particular, 67% (2/3) of them [46,56] used the Self-Assessment Manikin to investigate participants’ affective states across 3 dimensions: valence, arousal, and dominance. The Self-Assessment Manikin is characterized by a series of cartoonlike figures to represent different levels of each emotional dimension that participants should rate from pleasantness to unpleasantness. In this context, Messeri et al [67] used the Positive and Negative Affect Schedule to monitor changes in participants’ emotional states before and after experimental sessions. The Positive and Negative Affect Schedule includes 20 items, where 10 items assess positive affect and 10 items assess negative affect, both rated on a 5-point Likert scale.

#### Physiological Measures Used for Assessing Operators’ Affective Well-Being

The physiological measure that was most commonly used (6/46, 13% of the studies) was heart rate variability (HRV) for detecting stress symptoms through the analysis of heartbeats per minute [35,40,62,67,68,73]. In addition, 11% (5/46) of the studies used electrodermal activity to assess stress exertion by recording skin conductance levels, skin conductance responses, and galvanic skin response (GSR) [42,56,63,66]. Moreover, 4% (2/46) of the studies [44,45] relied on the skin potential response (SPR). SPR evaluates sweating as an indicator of sympathetic nervous system activity in human operators experiencing nervousness by detecting deviations of a few millivolts from the baseline electrical potential of the skin. Another 7% (3/46) of the studies used EEG signals to obtain brain data for evaluating anxiety symptoms [34,42,53].

Only 2% (1/46) of the studies relied on behavioral measures such as facial expression analysis to investigate anxiety symptoms in HCC through the use of Noldus FaceReader [52].

##### Measures Used for Assessing Cognitive Workload

#### Overview

Table 8 reports the distribution of self-report and physiological measures used for assessing cognitive workload.

**Table 8:** Measures used for assessing operators’ cognitive workload.

Measure			Studies, n (%)
**Self-report measures**			
	NASA^a^ Task Load Index	27 (59)	
	Situation Awareness Rating Technique	2 (4)	
**Physiological measures**			
	EEG^b^	1 (2)	
	Eye tracking	5 (11)	
	fNIRS^c^	2 (4)	
	HRV^d^	1 (2)	

^a^NASA: NASA.

^b^EEG: electroencephalography.

^c^fNIRS: functional near-infrared spectroscopy.

^d^HRV: heart rate variability.

#### Self-Report Measures Used for Assessing Operators’ Cognitive Workload

For evaluating cognitive workload, most studies (25/46, 54%) used the NASA Task Load Index (NASA-TLX) [32,33,35,37-39,41,45-49,51,53-55,59-62,64,66,70-72,74], with 8% (2/25) relying only on the frustration subscale [47,61]. Overall, the NASA-TLX is composed of 6 items that assess perceived workload across several dimensions: mental demands, physical demands, temporal demands, own performance, effort, and frustration. Each item is rated on a scale from 1 to 20. Moreover, 4% (2/46) of the studies [35,68] used the Situation Awareness Rating Technique to evaluate cognitive workload. The Situation Awareness Rating Technique consists of 10 items rated on a 7-point Likert scale. The measure assesses an individual’s perception of their situational awareness during task performance through 3 dimensions: demand on attentional resources, supply of attentional resources, and understanding of the situation.

#### Physiological Measures Used for Assessing Operators’ Cognitive Workload

A few studies (5/46, 11%) relied on eye-tracking tools [36,59,60,64,72] to measure gaze behavior and frequency and duration of fixations as indicators of cognitive workload, as well as to record pupil diameter and other eye-related biometric data to assess mental stress. In addition, 4% (2/46) of the studies used functional near-infrared spectroscopy [41,53] to measure neural hemoglobin dynamics and monitor cortical neural activations as indicators of cognitive workload. Finally, one study used HRV [65], and another study used EEG as an indicator of cognitive workload [53].

#### Key Findings Regarding Operators’ Affective Well-Being in Working With Cobots

##### Overview

As mentioned previously, well-being is typically investigated by assessing stress and anxiety symptoms. The interaction between participants and cobots showed varying impacts on stress and anxiety levels across different studies. In particular, the findings highlighted several factors that can affect stress and anxiety symptoms: the introduction of cobots within the workplace, the proximity of the cobot, the speed of the cobot manipulator, system usability, task complexity, the role of human operators, and the interaction scenarios.

With regard to stress symptoms, interestingly, Brun and Wioland [30] found that most operators felt less stressed and experienced reduced physical discomfort with the introduction of cobots within the workplace, viewing them as supportive tools. However, Mariscal et al [72] found no significant difference in stress levels when collaborating with a cobot versus another human operator, suggesting that the context of interaction may modulate the impact of cobots on stress levels.

##### Impact of the Physical Configuration of the Workspace and Cobot Behavior

Researchers observed that proximity to the cobot plays a crucial role in stress levels among operators. Arai et al [44] found that working closer to the cobot (1 m) significantly increased stress symptoms as measured using SPR, with participants displaying fear responses. Conversely, increasing the distance to 1.5 or 2 m seemed to alleviate these stress responses, highlighting the importance of the spatial configuration in the design of the workspace equipped with a cobot.

Furthermore, the speed of the cobot manipulator also critically affects stress levels. Fujita et al [45] and Gervasi et al [56] found that higher manipulator speeds (1000 mm/s) increased physiological stress indicators such as SPR and skin conductance level. Gualtieri et al [57] further observed that operator stress decreased when they were able to modify the cobot’s speed, indicating that providing operators with more control can reduce stress symptoms. This aligns with the findings of Pollak et al [73], who reported lower stress levels in manual control modes than in autonomous modes. Eyam et al [50] further demonstrated that cobots’ adaptive speed control in response to operator stress levels effectively reduced stress symptoms during task performance. Moreover, Messeri et al [67] noted that stress levels were highest when the cobot’s pace of interaction aimed solely to stimulate human productivity regardless of human stress levels. In addition, Dehais et al [69] reported that different cobot motions could affect stress levels, with certain motions eliciting higher GSRs. In particular, they tested 3 types of motions in a human-cobot object handover task. Motion 1 involved a human-aware planner with grasp detection and medium speed designed to be the safest and most legible and physically comfortable. Motion 2 had no planner, no grasp detection, and high speed, which was found to be the least legible, comfortable, and safe. Motion 3 used a planner without grasp detection and operated at a low velocity, which was moderately legible and safe, although it required the highest physical effort. The study showed that motion 2 elicited the highest GSR, indicating higher stress levels, whereas motion 1 elicited the lowest GSR, indicating lower stress levels. With regard to anxiety symptoms, Koppenborg et al [46] showed significant differences in anxiety levels based on cobot speed, with higher speeds increasing anxiety symptoms.

Not surprisingly, a good system usability impacts the operator’s stress levels. Specifically, communication interfaces between the cobot and operator significantly impact stress symptoms. In this regard, Arntz et al [47] found that augmented communication interfaces (eg, text panel, light signals, and gestures) reduced perceived stress and frustration compared to nonaugmented conditions in HCC. Amanhound et al [32] further emphasized that clear communication and timely feedback from cobots significantly reduced operator stress symptoms and enhanced task performance.

##### Impact of Task Configuration of the Workspace and Cobot Behavior

The complexity of tasks also plays a crucial role in determining stress levels. Zakeri et al [53] confirmed that task complexity notably impacted stress symptoms. Rajavenkatanarayanan et al [42] indicated that task timers and continuous work increased mental exertion and stress symptoms, whereas Brunzini et al [55] reported higher stress symptoms during phases requiring increased mental effort and additional stressors. Specifically, during phase 2 of their experimental protocol, participants experienced greater stress levels due to the dual-task nature of the activity, which combined the primary task of engine oil filter replacement with the secondary task of counting backward. This dual-task condition significantly increased the perceived mental workload and stress levels compared to phases in which tasks were performed without additional cognitive demands. Moreover, the presence of a time limit in phase 1 did not significantly impact emotional involvement, but phase 2 showed a 54% variation in stress levels, highlighting the substantial impact of the dual-task condition on stress symptoms and frustration. With regard to anxiety symptoms, Eimontaite et al [52] further indicated that task accuracy and signage tend to reduce anxiety symptoms.

##### Impact of Interaction Scenarios

Human operators’ roles and interaction scenarios further affect stress levels. Messeri et al [58] examined the impact of leader-follower dynamics in HCC focusing on physiological stress levels and productivity. In their study, the researchers assigned participants to 1 of 2 roles during a collaborative tower-building task: leaders, who directed the task, and followers, who complied with the cobot’s instructions. They found that followers in interaction with cobots exhibited higher stress levels than leaders, as evidenced by low- and high-frequency HRV and root mean square of successive differences metrics. Similarly, Lagomarsino et al [66] observed higher stress levels during cobot assistance than during human assistance, as indicated by hyperactivity and self-touching behaviors correlated with GSR recordings. Finally, Zhao et al [40] found that higher–task interdependence conditions, which require more coordinated effort between the human and the robot, led to increased stress and anxiety symptoms in human workers, although the evidence was not conclusive. The reciprocal interdependence condition, which necessitated the highest level of coordination and mutual adjustment, was particularly associated with these heightened mental states. This suggests that, while close collaboration with cobots can enhance task performance and efficiency, it also has the potential to increase the stress and anxiety levels of human operators due to the continuous need for coordination and the interdependent nature of the tasks.

#### Key Findings Regarding Operators’ Cognitive Workload in Working With Cobots

##### Overview

The analyzed studies revealed a nuanced understanding of how various factors and control strategies influence mental load and, concurrently, physical demands. In particular, several dimensions were identified that can affect the cognitive workload of operators working with cobots. These dimensions include force assistance in control strategies, the speed of the cobot, the impact of collaboration with cobots, design features, the complexity of the tasks, and unfamiliarity with the technology.

##### Impact of Force Assistance and Speed of the Cobot

One study [32] highlighted that incorporating force assistance in control strategies significantly reduced physical demands, as evidenced by the lower task load index scores compared to strategies without force assistance. This alleviation of physical demands also extends to reducing frustration and performance demands, underscoring the effectiveness of force-assisted modalities. Memar and Esfahani [34] found that high-damping setups are more suitable for tasks involving fine comanipulations as they tend to lower perceived workload, although researchers observed no significant differences in EEG-based workload predictions between high- and variable-damping setups.

Moreover, the cobot’s speed seems also related to cognitive workload. In particular, Fraboni et al [54] reported lower cognitive workload when participants could set the cobot’s speed, suggesting that user control over the cobot’s behavior can alleviate mental fatigue. Koppenborg et al [46] found a significant difference in mental workload between speed conditions, with higher scores in high-speed conditions. Similarly, Tan et al [43] found that mental workload increased with cobot motion speed and decreased with enhanced distance between humans and cobots. Van Dijk et al [75] reported that increased human autonomy and decreased cobot work pace significantly reduced cognitive demand and other workload factors.

##### Impact of Collaboration Types and Interaction Modalities

The impact of collaboration with cobots appeared to yield mixed results. Bettoni et al [62] showed a general reduction in both mental and physical workload when using a collaborative approach compared to a manual workstation. However, Fournier et al [70] reported no significant effect of collaboration with cobots on cognitive workload except for a positive effect on perceived time demand. In addition, results showed that working with cobots generated no greater mental load than working with humans based on the NASA-TLX results [72]. Moreover, Storm et al [31] found that participants spent more time in the focus zone when working with cobots compared to without and they often alienated themselves from the collaborative environment during these interactions.

With regard to the cobots’ design features, Pantano et al [48] noted that touch input interfaces as an interaction modality resulted in the lowest cognitive workload compared to speech-based interaction. In addition, Ustunel and Gunduz [74] found that, when the workstation equipped with a collaborative robotic arm integrated cognitive aids, the levels of operator workload decreased compared to the same workstation with no cognitive aid. The latter might include features such as augmented reality interfaces, intelligent decision support systems, and ergonomic setups that collectively reduce mental and physical strain on human operators. Not surprisingly, Panchetti et al [59] found that implementing usability guidelines, which ensure that interfaces are user-friendly and efficient, significantly reduced cognitive workload in different scenarios. Following this line, Rahman [37] found that better communication and smoother workflow with affective expressions reduced cognitive workload. Sadrfaridpour et al [39] also found that trust-based integrated frameworks resulted in the lowest perceived task load compared to manual and physical HRI–based methods. Lagomarsino et al [66] further observed that different interaction modalities significantly affected mental effort scores, whereas a greater transparency in cobot actions led to reduced cognitive demand. Lemasurier et al [33] found that motion-based signals such as feedback provided through movements and gestures were associated with the lowest cognitive workload scores, whereas without such feedback signals, cognitive workload levels increased.

Moreover, Aromaa et al [49] identified effort and frustration as key contributors to mental workload due to task complexity, for instance, when using a computer vision–based system to control the cobot. Brunzini et al [55] further observed higher mental demand and frustration in specific phases of their experimental protocol, such as phase 2, which involved executing a dual-task condition that required performing the primary task while simultaneously counting backward, whereas in phase 1, the stress levels were increased due to a time countdown, indicating that these trials required significant attention and concentration. Indeed, more complex activities conceived as dual tasks consistently led to increased mental load, as reported in several studies (4/46, 9%) [57,60,64,71]. Rossato et al [61] also reported higher frustration among older compared to younger workers in using manual control modalities as compared to touch screen modes. In this context, Sadrfaridpour and Wang [38] reported that conditions requiring manual control, where users had to directly manage and operate the system without automated assistance, resulted in significantly higher workload compared to assisted control conditions.

##### Impact of Task Complexity

In the study by Hopko et al [35], the findings suggest that task complexity can amplify the effects of fatigue, particularly on mental demand. The study highlighted that, under conditions of high mental demand induced by fatigue, female individuals reported greater difficulty in managing the cognitive load required for task performance. This effect was especially pronounced in more complex tasks. Similarly, Kalatzis et al [68] observed that sessions characterized by higher cognitive demands resulted in increased cognitive workload, as indicated by cardiovascular activity. Specifically, the study found that, during tasks with higher complexity, the low frequency–to–high frequency ratio in HRV was elevated, signifying greater cognitive effort. Finally, Luo et al [36] introduced an additional dimension to task complexity by highlighting the role of unfamiliarity with technology. They noted a conflict in results, observing that, while some previous research indicated that interacting with cobots in wholesale and retail environments increased cognitive workload and stress symptoms due to unfamiliarity with the technology and task complexity, their findings did not show significant differences in neurophysiological adaptation based on measures of muscle activity and pupil diameter.

#### Limitations of the Included Studies in HCC Research

The limitations across the included studies highlight several key areas that need improvements to enhance the generalizability and applicability of HCC research.

One significant limitation was the small sample sizes, as observed in several studies (19/46, 41%) [35,36,38,39,41,44-46,51,55,57-62,65-67].

Another common limitation was the homogeneity of participants’ demographics, particularly the prevalence of university students and staff members of the laboratory, as noted in several studies (21/46, 46%) [8, 35, 36, 38, 39, 41, 42, 45, 48, 49, 51, 57-62, 64-67].

Most studies (28/46, 61%) were conducted in controlled laboratory settings [34-36, 38, 39, 41, 42, 44-46, 48, 49, 51, 55-62, 64-67, 69-71].

In addition, task specificity was a recurring issue, with many studies focusing on specific tasks that may not represent realistic industrial activities or cover the full range of industrial applications. This limitation was evident in several studies (27/46, 59%) [34-36, 38, 39, 41, 42, 44-46, 48, 49, 52, 55-62, 64-67, 69, 71].

In numerous studies (21/46, 46%), the technology had a low technology readiness level, thereby reflecting issues related to its usability and maturity [35, 36, 38, 39, 41, 45, 46, 48, 49, 55-62, 64-66, 69].

Finally, because a large proportion of the studies (19/46, 41%) were short term, they provided limited insights into the long-term impact of HCC [35, 36, 38, 39, 41, 42, 45, 51, 56-62, 67, 69-71].

## Discussion

### Better Together: Factors Promoting Workers’ Well-Being and Balancing Cognitive Load in HCC

We systematically reviewed 46 studies published between 2009 and 2024, revealing significant trends and insights into the impact of cobots on human operators’ affective well-being and cognitive workload. Notably, there was a marked and constant increase in publications from 2020 onward, with a peak in 2023, likely reflecting the growing interest in understanding the human factors in HCC. Most studies (25/46, 54%) were conducted in Italy and the United States, primarily in laboratory settings and without involving real shop floor workers. Indeed, participants were mostly male university students or laboratory members aged approximately 29 years. This prevalence of controlled environments and nonrepresentative samples significantly limits the generalizability of the findings. Laboratory studies offer useful baseline insights, but they fail to capture the complexity, unpredictability, and organizational dynamics of real-world industrial settings. In actual workplaces, factors such as shift patterns, production pressure, diverse skill levels, and sociotechnical constraints may interact with cobot integration in ways that are not observable in laboratory conditions. Moreover, real-world HCC is embedded within social structures in which workers must navigate interpersonal relationships with managers and colleagues [21]. Cobot adoption may influence these dynamics, affecting perceptions of fairness, trust, role clarity, and social cohesion on the shop floor. For example, workers might experience increased stress not only from the cobot itself but from unclear expectations, fears of job replacement, or tensions in team collaboration [21].

Moreover, the most used advanced robotic system was Universal Robots through different weight categories used particularly for assembly and disassembly tasks, thereby failing to reflect the variety of robotic systems actually deployed in real-world settings.

In particular, the included studies primarily focused on exploring aspects such as cognitive or mental workload, human factors, and occupational safety using physiological measurements and self-report tools either alone or combined. Researchers have paid considerably less attention to emotions, affective states, well-being, and symptoms of anxiety and stress. It is worth noting that none of the included studies examined depression symptoms, which are critical indicators of chronic stress [78-80]. This imbalance highlights the necessity for future research to explore the emotional and psychological aspects of working with cobots more thoroughly. By understanding these dimensions, companies can develop strategies that enhance the affective well-being of human operators while simultaneously maintaining or improving productivity and efficiency. This, in turn, can inform the design and implementation of cobot systems that not only support operational goals but also foster a healthier, more supportive work environment for operators.

In terms of affective well-being, the studies observed that, while cobots have been shown to alleviate physical fatigue and improve job satisfaction, they also introduce new psychological challenges. These include stress and anxiety symptoms stemming from concerns about job security and the stress that comes with high-paced operations. Critical factors influencing these outcomes include the modality of the introduction of cobots within the workplace, the proximity of cobots, the speed at which cobots operate, the system usability, and the complexity of tasks assigned. In particular, Brun and Wioland [30] found that operators experienced fewer stress symptoms and physical discomfort when cobots were newly introduced. Moreover, operators’ stress levels increased significantly when cobots operated at higher speeds or in close proximity [44-46,56,57,61,62,69]. In this regard, findings may suggest that limiting the manipulator speed to lower levels, preferably <500 mm/s, could mitigate stress symptoms and enhance operator comfort. Indeed, providing operators with the ability to control cobot behavior, such as adjusting speeds, can mitigate these stress responses and enhance overall comfort [57]. In addition, effective system usability significantly impacts operator stress levels, particularly through communication interfaces between cobots and human operators. Arntz et al [47] showed that augmented interfaces (eg, text panels, light signals, and gestures) decreased perceived stress and frustration, whereas Amanhound et al [32] highlighted that clear communication and timely feedback from cobots further alleviated stress and enhanced task performance. In this regard, it is also important to consider the task complexity, which significantly impacts stress [53,55] and anxiety levels. For instance, Rajavenkatanarayanan et al [42] highlighted that the complexity introduced by task timers and continuous work heightened stress symptoms. Eimontaite et al [52] noted that task accuracy and signage reduced anxiety symptoms.

In terms of cognitive workload, studies found similar results, pointing out that higher task complexity increased cognitive load, with dual-task conditions significantly heightening mental workload compared to single-task conditions [36,38,49,55,57,59-61,64,71]. Moreover, studies indicated that user control over cobot behavior, such as adjusting speed, alleviated mental fatigue [54,75], whereas high-speed conditions increased mental workload [43,46]. Furthermore, collaborating with cobots generally reduced both mental and physical demands compared to manual workstations [62], although the cobots’ effect on participants’ cognitive workload remained mixed [70]. Collaboration with cobots did not generate greater mental load compared to human collaboration [72]. However, it can make users feel alienated despite increased focus time [31]. In this regard, effective communication interfaces and adaptive systems that adjust cobot actions based on real-time assessments of human stress levels and cognitive load are promising strategies to improve HCC quality and, thus, their fluency, meaning the smoothness and quality of their interaction. Indeed, studies highlighted that integrating cognitive aids [74], usability guidelines [59], affective communication [37], trust-based frameworks [39], interaction modalities [48,65,66], and motion-based signals [33] all contributed to lower cognitive workload. Another important aspect that emerged from this systematic review concerns the considerable heterogeneity in how affective well-being was measured across the studies. Tools ranged from validated self-report scales to physiological proxies such as HRV. The lack of consensus on which instruments best capture affective responses to HCC hinders replication and slows cumulative progress in the field. To address this issue, future research should focus on the development and adoption of a standardized, multidimensional battery for assessing affective well-being in HCC contexts. This could include widely used and validated psychometric tools (eg, the 7-item Generalized Anxiety Disorder scale for anxiety, the 9-item Patient Health Questionnaire for depression symptoms, and the PSS for perceived stress) supplemented by context-sensitive physiological measures and qualitative interviews to triangulate findings and ensure robustness.

### Limitations and Future Developments in Research and Real-World Settings

As this is the first systematic review specifically focused on the affective well-being and cognitive workload of operators in HCC, it was crucial to first establish a comprehensive narrative mapping of the existing evidence. This foundational step aligns with the best practices recommended for early-stage systematic reviews in nascent research domains [76]. While a meta-analysis can provide statistical rigor and pooled effect estimates, it was not feasible to conduct one in the context of this review. The motivation was primarily the high degree of heterogeneity across the included studies, particularly in terms of study designs, participant populations, settings (eg, controlled laboratories vs industrial environments), measures used (eg, NASA-TLX, HRV, EEG, and various self-report scales), and outcomes reported. In addition, many studies reported incomplete or nonstandardized statistical data, such as missing effect sizes or inconsistent outcome reporting, which further limited the feasibility of a quantitative synthesis. This limitation is consistent with what has been noted in other systematic reviews in emerging interdisciplinary fields, where methodological variability and limited high-quality data preclude meaningful meta-analytical aggregation [77,81]. This systematic review highlights that cobots offer substantial benefits in terms of productivity and safety in manufacturing but also that they undoubtedly weigh on the workers’ mental workload, especially when the situational complexity is high. However, the fact that nearly all studies (44/46, 96%) were set in controlled laboratory settings and with the involvement of nonrepresentative participants and tasks highlights the need to conduct experiments in more ecological conditions (ie, in industrial settings). This would help research better reflect the diverse characteristics and competences of workers and the full range of activities they perform. Real-world scenarios will pose significant challenges to the robustness of physiological measurements, which were frequently used in the reviewed studies. To overcome this, future research may integrate other research methods (eg, video analysis, as done in the study by Kopp et al [82] to assess mental workload in a manual assembly task). Future research should also broaden its scope to include the evaluation of depressive symptoms alongside anxiety and stress symptoms as all are critical indicators of operators’ affective well-being. Addressing these psychological factors using similar standardized measures can significantly enhance overall affective well-being, leading to increased productivity in the organization. Moreover, the addition of a qualitative approach (eg, interviews) would allow for better understanding of the workers’ experience and would also compensate for the substantial lack of qualitative methods in the retrieved studies. Studying cobots in real-world settings would also make it possible to evaluate those with a high technology readiness level, which are technological solutions that can actually be deployed in manufacturing.

Consistently with the paradigm of Industry 5.0, these systems should be designed placing the human operator at the forefront. To this end, workers should be enabled to control the features of the cobots’ movements whenever possible. In addition, we recommend that manufacturing companies integrate cobots gradually involving workers throughout the process and using pilot phases that include both technical training and psychological monitoring of workers, as already highlighted in the study by Kopp et al [82], given the proven impact that interacting with cobots can have on workers’ cognitive and affective well-being. Human resources departments are encouraged to include mental health assessments in routine occupational evaluations to monitor the fitness of the HCC and, eventually, be able to promptly address emerging issues. In addition, interdisciplinary collaboration between engineers, psychologists, and safety officers should be institutionalized to co-design adaptive cobot systems that account for the best interests of both organizations and workers. These strategies will help transform technological innovation into sustainable workplace practices that align with the human-centered goals of Industry 5.0. Finally, research efforts should be focused on developing systems capable of dynamically adjusting cobot behaviors in response to human operators’ stress and anxiety symptoms as well as cognitive workload. This approach will support the physical aspects of HCC while also prioritizing the operators’ affective well-being.

### Conclusions

This systematic review highlighted key indicators related to the impact of cobots on the affective well-being and cognitive workload of human operators. The analysis of these studies revealed that, while cobots alleviate physical fatigue and improve job satisfaction, they also introduce new psychological challenges, such as increased stress and anxiety symptoms, particularly during high-speed and proximity operations. In addition, increased task complexity and dual-task conditions significantly increase cognitive workload. Furthermore, the results of these studies emphasized the potential of adaptive systems, which allow for the real-time regulation of cobots’ behavior and provide operators with more control as these systems can significantly reduce stress symptoms and cognitive workload, thereby improving overall affective well-being and efficiency. Notwithstanding these promising results, the generalizability of these findings is limited by small, homogeneous samples and controlled laboratory environments. Therefore, future research should address these limitations by diversifying participant samples and conducting studies in real industrial environments to improve ecological validity.

In addition, the consistent use of the NASA-TLX to assess cognitive workload in all these studies ensured comparability of results, demonstrating a clear trend of increasing cognitive demands with more complex tasks. However, the assessment of affective well-being was less homogeneous, with the adoption of a wide variety of measures, which makes it difficult to compare results across studies and underlines the need for more consistent assessment tools in this domain.

Therefore, it is crucial to standardize the use of these measures as it will potentially improve methodological rigor and facilitate comparative analyses between studies. In this regard, the combined use of physiological measures and self-report tools to comprehensively assess stress, anxiety, and depressive symptoms along with cognitive and physical workload assessments is crucial. Furthermore, there is the need to integrate qualitative methodologies that provide insights into the lived experiences of operators working with cobots. By capturing the subjective experiences of these operators, the broader implications of the use of cobots in manufacturing can be better understood.

Mindful of this, a holistic approach to well-being is essential. This approach recognizes that well-being includes not only the alleviation of symptoms of stress and anxiety but also the management and reduction of depressive symptoms and other crucial psychological factors. For example, it is necessary to comprehend the individual as a complex human being, including the emotional, mental, and social dimensions. By addressing these aspects, it is possible to develop a more nuanced understanding of the psychological impact of cobots on human operators and subsequently implement strategies to more effectively support the affective well-being and cognitive workload of operators working with cobots.

Future research should aim to develop longitudinal studies that track psychological responses to cobots over time, ideally in real industrial environments rather than controlled laboratory settings. Samples should include diverse worker populations such as shift workers, older adults, and nontechnical staff to improve ecological validity and generalizability. Mixed methods approaches that combine quantitative measures (eg, HRV, EEG, and NASA-TLX) with qualitative methods (eg, interviews) will be critical to fully capture the complexity of HCC. In addition, experimental designs that manipulate variables such as cobot autonomy, task complexity, or interface transparency can help isolate causal effects on cognitive workload and affective well-being.

Therefore, the development of these strategies will create healthier and more supportive work environments and refine the design and implementation of cobot systems, thus ensuring greater efficiency and productivity.

## Appendix

Multimedia Appendix 1 Final search strategy.

Multimedia Appendix 2 PRISMA checklist.

## Abbreviations

EEG: electroencephalography
GSR: galvanic skin response
HCC: human-cobot collaboration
HRC: human-robot collaboration
HRI: human-robot interaction
HRV: heart rate variability
NASA-TLX: NASA Task Load Index
PRISMA: Preferred Reporting Items for Systematic Reviews and Meta-Analyses
PSS: Perceived Stress Scale
SPR: skin potential response
UI: user interface
VR: virtual reality
