# Supplementary material for: Understanding Workers’ Well-Being and Cognitive Load in Human-Cobot Collaboration: Systematic Review
Source: J Med Internet Res. 2025 Aug 27;27:e75658. doi: 10.2196/75658 (PMC12423613; doi:10.2196/75658)
Supplement: Multimedia Appendix 1 [file jmir_v27i1e75658_app1.docx]

This document provides the final search strategies used for each database included in the systematic review. Searches were carried out up to April 2024. Boolean operators were used where applicable to ensure comprehensive coverage. Search strategies may vary slightly in syntax based on the database’s query structure.

**Web of Science**

TS=("cobot*" OR "collaborative robot*" OR "human-robot interaction" OR "human-cobot collaboration") AND TS=("manufacturing") AND TS=("well-being" OR "stress" OR "anxiety" OR "depression") AND TS=("cognitive workload" OR "mental workload")

**Scopus**

TITLE-ABS-KEY("cobot*" OR "collaborative robot*" OR "human-robot interaction" OR "human-cobot collaboration") AND TITLE-ABS-KEY("manufacturing") AND TITLE-ABS-KEY("well-being" OR "stress" OR "anxiety" OR "depression") AND TITLE-ABS-KEY("cognitive workload" OR "mental workload")

**ACM Digital Library**

("cobot*" OR "collaborative robot*" OR "human-robot interaction" OR "human-cobot collaboration") AND "manufacturing" AND ("well-being" OR "stress" OR "anxiety" OR "depression") AND ("cognitive workload" OR "mental workload")

**IEEE Xplore**

("cobot*" OR "collaborative robot*" OR "human-robot interaction" OR "human-cobot collaboration") AND ("manufacturing") AND ("well-being" OR "stress" OR "anxiety" OR "depression") AND ("cognitive workload" OR "mental workload")
